# Spontaneous resolution of traumatic pseudoaneurysm: A case report

**DOI:** 10.1097/MD.0000000000046184

**Published:** 2025-11-21

**Authors:** Jiajie Gu, Jianru Li, Yongzhi Zhao

**Affiliations:** a Department of Neurosurgery, People’s Hospital Affiliated of Ningbo University, Ningbo, Zhejiang, China; b Department of Neurosurgery, The Second Affiliated Hospital of Zhejiang University Medical College, Hangzhou, Zhejiang, China; c Department of Neurosurgery, Haining Central Hospital, Jiaxing, Zhejiang, China.

**Keywords:** case report, spontaneus resolution, traumatic pseudoaneurysm

## Abstract

**Rationale:**

A traumatic pseudoaneurysm (TP) is a rare vascular pathology. Once diagnosed, active surgical intervention is usually required. However, in addition to growth and rupture, the natural outcomes of TPs also include spontaneous resolution. This article reports a rare case of a TP that resolved spontaneously, with the aim of informing future clinical strategies.

**Patient concerns:**

A 47-year-old female patient underwent decompressive craniectomy with hematoma evacuation following head trauma. Initial computed tomography angiography demonstrated a pointed protrusion along the ophthalmic segment of the left internal carotid artery. Two weeks post-injury, follow-up computed tomography angiography revealed a saccular protrusion at the same site, measuring 6.0 mm × 3.7 mm with a neck width of 6.3 mm. Digital subtraction angiography (DSA) at 3 weeks post-injury confirmed a saccular aneurysm in the ophthalmic segment of the left internal carotid artery.

**Diagnoses:**

The combination of a history of trauma and a rapidly growing lesion was highly suggestive of a TP.

**Interventions:**

Four weeks post-injury, follow-up DSA demonstrated a progressive reduction in the aneurysm size, prompting the family to opt for conservative management.

**Outcomes:**

Eight weeks post-injury, DSA confirmed the spontaneous disappearance of the aneurysm. A 1-month post-discharge telephone follow-up revealed no symptoms of clinical recurrence, such as headache or epistaxis.

**Lessons:**

Since spontaneous healing of TP is uncommon and its mechanism remains unclear, conservative management mandates close imaging surveillance, as a risk of recurrence persists even with the most prudent patient selection.

## 1. Introduction

Traumatic aneurysms account for approximately 1% of intracranial aneurysms and are typically located near fracture sites or where arteries are closely adherent to the dura mater.^[1,2]^ Intracranial arteries are muscular-type arteries with a lack of elastic fibers in the outer membrane, making them susceptible to rupture and hemorrhage due to external force.^[3,4]^ Following the liquefaction of extramural hematomas, the cavitated blood clot replaces the original vascular wall. Consequently, these lesions are classified as pseudoaneurysms.^[5–7]^

Traumatic pseudoaneurysms (TPs), due to the absence of a normal aneurysmal wall structure, are characterized by irregular morphology, rapid growth over a short period, and a high risk of rebleeding.^[8]^ Once diagnosed, active surgical intervention is usually required. Common approaches include endovascular treatment and craniotomy. However, in addition to growth and rupture, the natural outcomes of TPs also include spontaneous resolution.

In this article, we report a rare case of a TP that resolved spontaneously.

## 2. Case report

### 2.1. Case details

**Figure 1.**
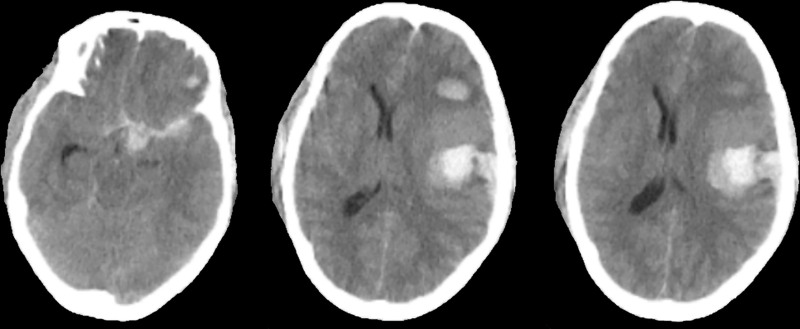
Brain axial computed tomography (CT) scan of the patient prior to surgery.

**Figure 2.**
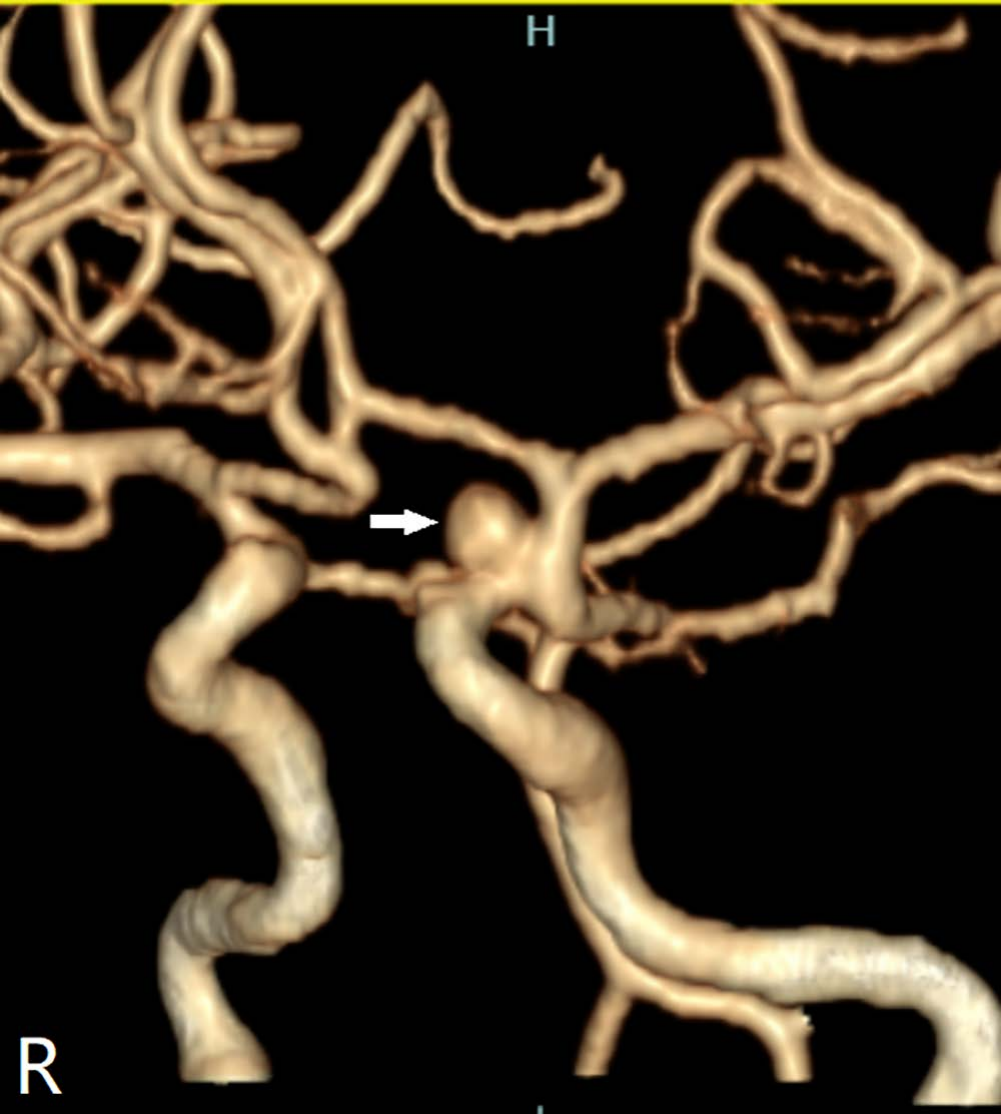
Computed tomography angiography (CTA) performed at 2 weeks post-injury showing a left internal carotid artery (ICA) ophthalmic aneurysm (arrowhead).

**Figure 3.**
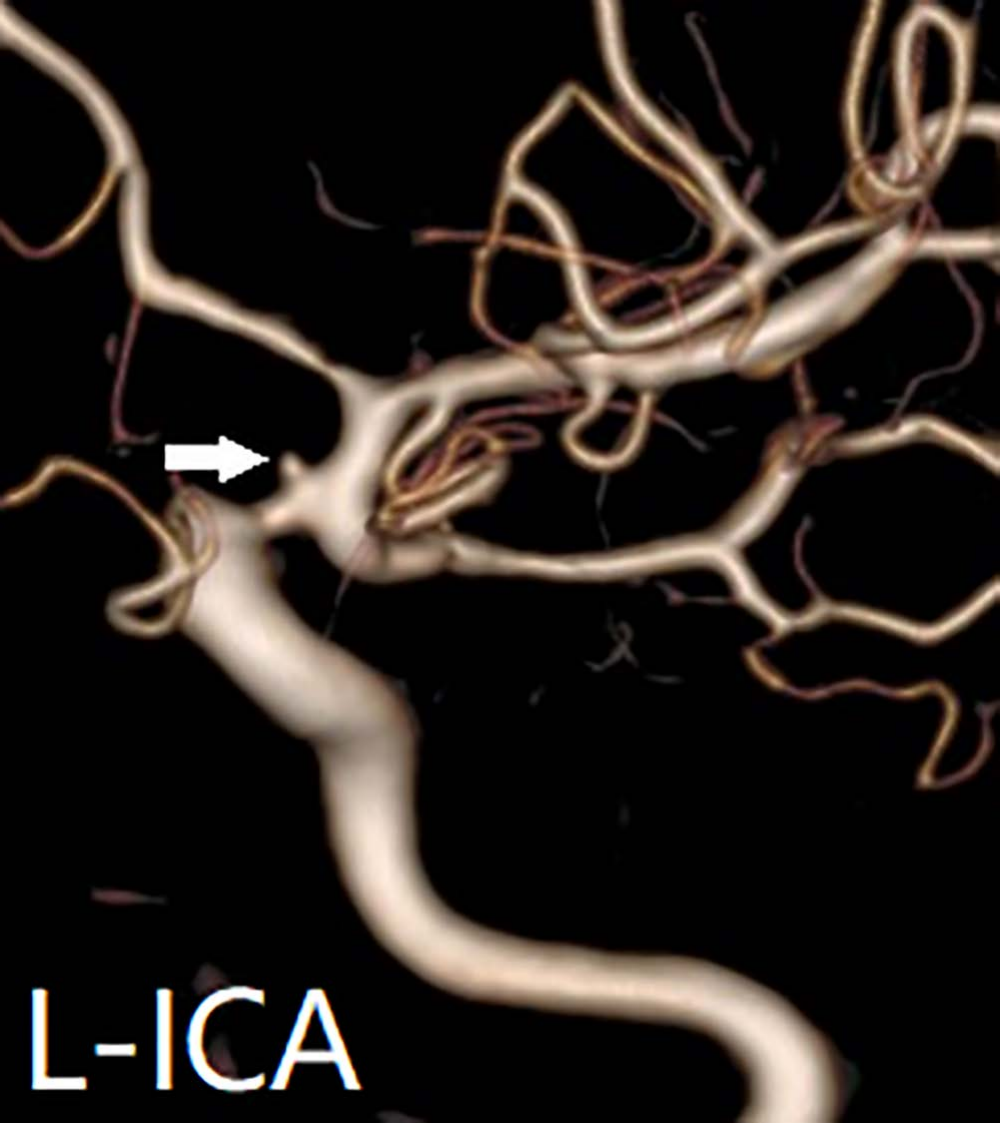
Three-dimensional digital subtraction angiography (3D-DSA) taken at 3 weeks post-injury showing a saccular aneurysm measuring 1.6 mm at the neck and 1.6 mm in diameter in the left internal carotid artery (ICA) ophthalmic segment (arrowhead).

**Figure 4.**
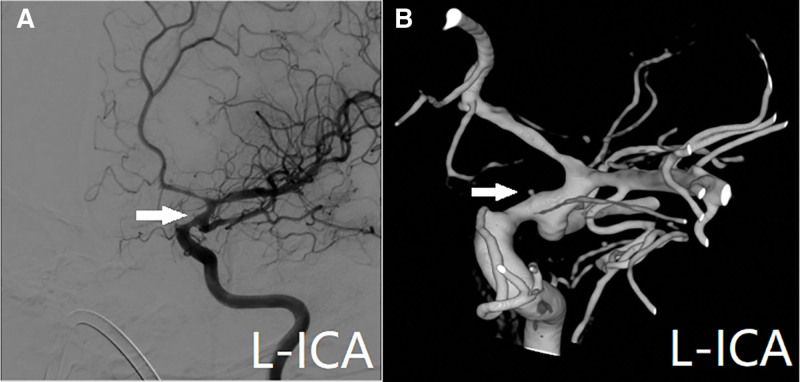
Digital subtraction angiography (DSA) image at 4 weeks post-injury, (A) lateral view DSA and (B) 3-dimensional DSA (3D-DSA) showed a left internal carotid artery (ICA) ophthalmic aneurysm with 0.73 mm in diameter (arrowhead).

A 47-year-old female presented to a local hospital on October 28, 2024, due to head trauma. She had a medical history of diabetes mellitus and had been on long-term metformin therapy. Computed tomography (CT) revealed a left frontotemporoparietal hematoma with associated subarachnoid hemorrhage, accompanied by fractures of the right temporal and sphenoid bones (Fig. 1). CT angiography (CTA) showed a pointed protrusion in the left internal carotid artery (ICA) ophthalmic segment, with a suspected arterial cone. Emergency decompressive craniectomy with hematoma evacuation was performed. Two weeks post-injury, follow-up CTA revealed a saccular protrusion at the same site, measuring 6.0 mm × 3.7 mm with a neck width of 6.3 mm (Fig. 2; data sourced from local hospital radiographic reports). Three weeks post-injury, digital subtraction angiography (DSA) demonstrated a saccular aneurysm in the left ICA ophthalmic segment, with a neck width of 1.6 mm and dimensions of 1.3 mm × 1.6 mm (Fig. 3). Based on the patient’s history of trauma and the characteristic rapid growth of the lesion over a short period, a TP was diagnosed. Flow diversion stent placement was recommended, following which the patient was transferred to our institution. Physical examination on admission: comatose with a left cranial defect. The Glasgow Coma Scale score was 11/15. Four weeks post-injury, follow-up DSA showed a reduction in the aneurysm size, with a neck width of 0.55 mm and a size of 0.71 mm × 0.73 mm (Fig. 4). Given the progressive reduction in aneurysm size and the inherent hemorrhagic risk of antiplatelet therapy, coupled with the patient’s lack of a hypertension history and stable hemodynamics during hospitalization, a strategy of close monitoring was recommended. Definitive surgical repair was to be actively undertaken once growth is confirmed. The family was explicitly informed about the hemorrhagic risk associated with conservative management, even in the setting of aneurysm size reduction. After thorough communication, the family demonstrated full understanding and trust, and opted for continued observational management.

### 2.2. Results and follow-up

**Figure 5.**
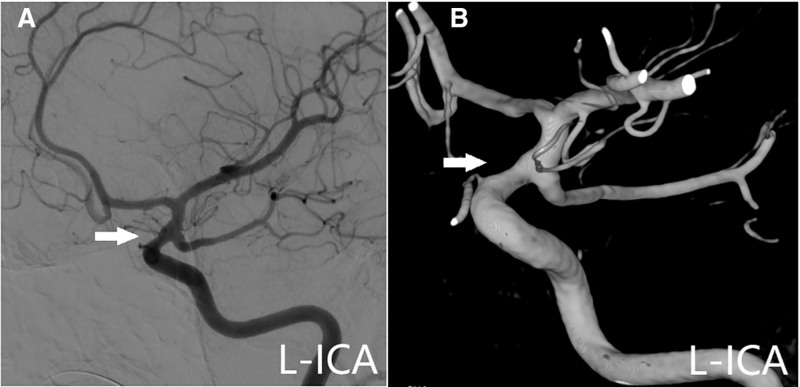
Cerebral angiogram, 8 weeks post-injury, (A) lateral view digital subtraction angiography (DSA) and (B) 3-dimensional DSA (3D-DSA) demonstrated spontaneous resolution of the pseudoaneurysm (arrowhead).

**Figure 6.**
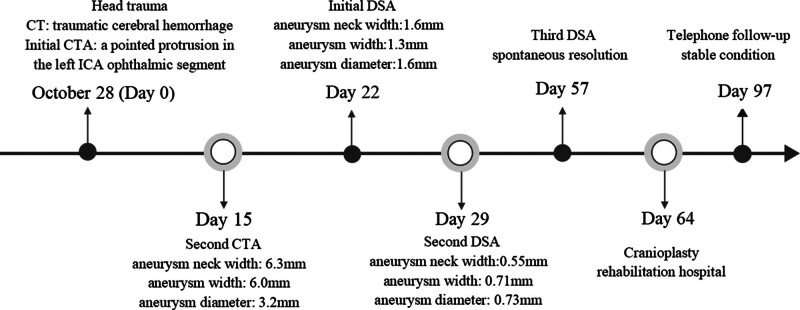
Diagnosis and treatment flowchart. CT = computed tomography, CTA = computed tomography angiography, DSA = digital subtraction angiography, ICA = internal carotid artery.

Eight weeks post-injury, follow-up DSA revealed no significant aneurysm visualization (Fig. 5). Nine weeks post-injury, the patient regained consciousness and was discharged for rehabilitation following a left cranioplasty. There was no significant change in the latest head CT. A 1-month post-discharge telephone follow-up revealed no symptoms of clinical recurrence, such as headache or epistaxis. The complete treatment process is illustrated in Figure 6.

### 2.3. Study protocol and image analysis

Throughout the entire treatment course, no antiplatelet or anticoagulant agents were administered. Angiography was performed using an Allura Clarity or Azurion Clarity IQ system (Philips Healthcare, Amsterdam, The Netherlands) equipped with a digitally linked 3-dimensional rotational angiographic (3D-RA) reconstruction workstation (Xtravision, Philips Healthcare). The 3D-RA acquisition protocol was as follows: rotation angle: 240°; rotation time: 4.0 seconds; frame rate: 6 fps; injection delay: 2 seconds; field of view: 42 cm.

DSA image analysis was performed independently by 2 neurosurgeons, each with at least 15 years of neurointerventional experience, using the Philips Allura 3D-RA workstation to identify the location and dimensions of the lesion. The measured parameters were defined as follows: aneurysm neck: the maximum diameter of the cross-section at the neck level; aneurysm height: the maximum distance perpendicular from the neck plane to the dome; maximum aneurysm diameter: the greatest distance from the midpoint of the neck to the dome; and aneurysm width: the longest segment perpendicular to the maximum diameter intersecting the aneurysm wall. The average values across all parameters were used as the final results.

This case report was approved for publication by the Institutional Review Board of Haining Central Hospital, and written informed consent was secured from the family.

## 3. Discussion

TPs are a rare vascular complication following craniocerebral injuries, most commonly occurring in the cavernous or petrous segments of the ICA, often caused by skull base fractures. According to reports, TPs typically form and rupture 2 to 3 weeks after the injury.^[9]^ Clinically, vascular imaging is necessary for patients with the following characteristics: skull base fractures accompanied by severe subarachnoid hemorrhage; recurrent subarachnoid hemorrhage post-injury or hematomas at the skull base; repeated episodes of delayed epistaxis. Due to their small size and unique morphology, TPs can be challenging to diagnose on CTA or DSA. Palmieri et al suggest that even if the initial angiographic results are negative, follow-up examinations should be conducted.^[10]^ A second DSA should be performed 3 weeks after the trauma, with additional follow-up at 6 to 12 weeks if necessary.^[11]^

Currently, there is no uniform diagnostic criterion for TPs. On imaging, TPs often present as a sac-like outpouching from the arterial wall. The key differential diagnoses include fibromuscular dysplasia, dissecting aneurysms, and mycotic pseudoaneurysms. Fibromuscular dysplasia predominantly affects young women and is characterized by the pathognomonic “string of beads” appearance in the carotid artery.^[12]^ DSA of a dissecting aneurysm typically reveals the string sign, the double lumen sign, aneurysmal dilatation, and contrast pooling.^[13]^ Mycotic pseudoaneurysms are more predisposed to a saccular, eccentric, or lobulated configuration and are often accompanied by clinical evidence of sepsis or a local infection.^[14,15]^ Pathologically, aneurysms arising from the anterior wall of the ICA can be divided into 2 categories: saccular aneurysms and blood blister-like aneurysms (BBAs). The clinical diagnosis of BBAs is primarily based on the criteria proposed by Fang et al, which are as follows: aneurysms located at the supraclinoid ICA projecting anteriorly; nonbranching sites; initially small (maximum diameter <10 mm); subarachnoid hemorrhage corresponding to the aneurysm; rapid growth (<2 weeks) on repeated angiograms (CTA or DSA); and an irregular wall of the aneurysm or of the parent artery. An aneurysm is diagnosed as a BBA of the ICA when criteria 1 to 4 are all matched, and either criterion 5 or 6 is matched as well.^[16]^ Based on the aforementioned diagnostic criteria, imaging characteristics, and the history of head trauma, there was a high clinical suspicion for a BBA. However, the diagnostic gold standard for BBA is intraoperative findings during craniotomy, not imaging.^[17]^ In the absence of histopathological evidence, this case was prudently diagnosed as a TP.

TPs are characterized by the absence of a true aneurysmal wall and a neck and carry a high risk of rebleeding. In principle, surgical intervention should be performed as early as possible once the diagnosis is confirmed.^[18]^ The key to the procedure lies in reducing the risk of rebleeding while simultaneously minimizing stenosis of the parent artery to ensure adequate distal blood supply. The main surgical approaches include aneurysm clipping and trapping combined with bypass surgery. For patients who require aneurysm trapping, parent artery occlusion, and extracranial-intracranial bypass, a preoperative Matas test can aid in acclimatization to cerebral ischemia. With rapid advances in interventional materials and techniques, endovascular treatment has emerged as a pivotal modality for managing TPs. The primary endovascular techniques include stent-assisted coiling, endovascular parent artery occlusion, covered stents, and flow diversion. It is crucial to note that due to the deficient vascular wall structure of TPs, stand-alone coil embolization is prone to coil migration and aneurysm recurrence.^[19]^ Therefore, this option is generally not recommended.

In this case, we observed the spontaneous healing of a TP. According to existing literature, this condition has been commonly documented in cases such as ruptured aneurysms,^[20]^ dissecting aneurysms,^[21]^ following revascularization procedures for moyamoya disease,^[22]^ and arteriovenous malformations posttreatment.^[23]^ However, it is rarely observed in TPs. To our knowledge, 7 similar cases have been reported in the literature,^[24–30]^ with 4 located in the external carotid artery,^[24–27]^ 1 in the anterior circulation,^[30]^ and 2 in the posterior circulation.^[28,29]^ With the exception of 1 case that recurred in the anterior circulation,^[30]^ the remaining 6 cases resolved completely (Table 1). Knowledge regarding TPs is limited since it is based on a small number of cases and literature reviews. In a 2021 systematic review, Palmieri et al aimed to elucidate the features, natural history, and management of traumatic posterior circulation pseudoaneurysms. Despite this effort, the rarity of the condition limited the final inclusion to just 34 cases.^[10]^ In contrast to previous reports, our case more comprehensively delineates the dynamic process of a TP, from formation to spontaneous disappearance.

**Table 1:** Cases of spontaneous resolution of unruptured pseudoaneurysms.

References	Age (yr)/Sex	Location	Pathogeny	Time to resolution	Obliteration outcome	Post-resolution follow-up	Recurrence
Raskin et al^[24]^	80/F	Superficial temporal artery	Traumatic	9 mo	Complete disappearance	9 mo	No
Atiles et al^[25]^	42/M	Middle meningeal artery	Traumatic	11 mo	Complete disappearance	NA	NA
Srinivasan et al^[26]^	25/M	Middle meningeal artery	Traumatic	2 wk	Complete disappearance	10 wk	No
Shah et al^[27]^	39/M	Middle meningeal artery	Traumatic	1 mo	Almost complete disappearance	NA	NA
Moron et al^[28]^	6/M	Posterior cerebral artery	Traumatic	15 d	Complete disappearance	5 yr	No
Tekiner et al^[29]^	65/F	Vertebral artery	Traumatic	2 yr	Complete disappearance	NA	NA
Zanaty et al^[30]^	55/M	Anterior choroidal artery	Iatrogenic injury	3 d post-operation	Complete disappearance	1 wk	Yes

F = female, M = male, N/A = not available.

Most researchers attribute the radiological regression of such aneurysms primarily to thrombosis.^[28,31]^ Moron et al reported a case of a 6-year-old boy who developed a posterior cerebral artery aneurysm after a head injury. Serial imaging over a 5-year period demonstrated progressive reduction and calcification of the thrombosed aneurysm.^[28]^ Cai et al also detected thrombus formation within spontaneously healed ruptured aneurysms using high-resolution magnetic resonance imaging (HR-MRI).^[31]^ Studies showed that the large size of giant cerebral aneurysms promotes thrombus formation by inducing slow and turbulent flow.^[32,33]^ Although small unruptured aneurysms lack morphological features associated with thrombosis, they may still contribute to ischemic events.^[34]^ A hemodynamic study conducted by Ribeiro et al identified a shear rate threshold below which thrombus occurs within the aneurysm sac.^[35]^ However, the precise mechanism driving thrombogenesis within aneurysms remains poorly defined. It is important to note that Vandenbulcke et al found that thrombosis is not a definitive marker of aneurysm healing, as it may be associated with vascular recanalization, rupture, or ischemic events.^[33]^

Spontaneous thrombosis alone cannot fully explain the mechanism of spontaneous healing in TPs. Chow et al reported a case of a 43-year-old female patient who underwent endovascular treatment for an ICA aneurysm. A concurrent distal small aneurysm, which was managed conservatively, was found to have resolved completely on a DSA performed 10 weeks after the procedure. The authors proposed that this resolution was attributed to hemodynamic changes following the embolization.^[36]^ Enomoto et al similarly documented the spontaneous resolution of a ruptured basilar artery perforator aneurysm.^[37]^ Cohen et al postulated that this phenomenon might be associated with hypotension, vascular spasm, and damage to the aneurysm wall.^[32]^ In comparison with our case, the underlying pathological mechanisms appear to differ among the reported cases.

In a study by Sakata et al, it was observed that following aneurysmal rupture, the injured vascular wall demonstrated proliferation of smooth muscle cells, accumulation of macrophages, and infiltration of lymphocytes, indicating a persistent vascular remodeling process.^[38]^ In the histological specimens from a spontaneous superficial temporal artery pseudoaneurysm, Takemoto et al identified atherosclerosis with intimal thickening and medial calcification. Atherosclerotic plaque impairs the elasticity of the vascular intima, rendering the vessel wall more susceptible to dissection.^[39]^ Therefore, we hypothesize that the spontaneous healing of TPs may be associated with the absence of atherosclerotic plaque, thrombus formation, hemodynamic stability, and vascular remodeling. On review of the case, the patient’s long-term metformin therapy and lack of hypertensive history suggested the eventual outcome. A stable hemodynamic status promotes a low-shear environment for the vascular wall, thereby preventing further aneurysmal growth. Simultaneously, sluggish blood flow is favorable for thrombus formation at the rupture site. Moreover, metformin has been shown to have anti-inflammatory,^[40]^ endothelium-protective,^[41]^ and anti-atherosclerotic properties.^[42]^ These factors collectively conspire to promote spontaneous healing of the aneurysm. Unfortunately, in this case, HR-MRI of the vessel wall was not performed.

Unlike that of TPs, the spontaneous disappearance of iatrogenic pseudoaneurysms can be deceptive. Zanaty et al reported a case of a 55-year-old man who developed an anterior choroidal pseudoaneurysm due to arterial injury during pituitary tumor surgery. The pseudoaneurysm disappeared on the second angiogram but reappeared 1 week later, ultimately requiring treatment with a flow-diversion device.^[30]^

This case has some limitations. First, the generalizability of the conclusions is limited by the single-case design. Second, HR-MRI of the vessel wall was not performed, which could have provided additional insight into the healing mechanism. Third, no subsequent CTA or DSA was performed after the aneurysm resolution, and long-term follow-up was lacking. The natural history of TPs remains unclear. While spontaneous healing is rare, close clinical follow-up is still warranted even after lesion disappearance.

## 4. Conclusion

Since spontaneous healing of TP is uncommon and its mechanism remains unclear, conservative management mandates close imaging surveillance, as a risk of recurrence persists even with the most prudent patient selection. Future large-scale, multicenter studies are needed to further clarify the potential factors contributing to spontaneous resolution.

## Acknowledgments

The authors thank the patient and her family for providing consent to present this case.

## Author contributions

**Conceptualization:** Jiajie Gu.

**Data curation:** Jiajie Gu.

**Project administration:** Jianru Li.

**Supervision:** Yongzhi Zhao.

**Validation:** Yongzhi Zhao.

**Writing – original draft:** Jiajie Gu.

**Writing – review & editing:** Jianru Li, Yongzhi Zhao.
